# The Structure of Conscious Bodily Self-Perception during Full-Body Illusions

**DOI:** 10.1371/journal.pone.0083840

**Published:** 2013-12-23

**Authors:** Martin Dobricki, Stephan de la Rosa

**Affiliations:** Max Planck Institute for Biological Cybernetics, Tuebingen, Germany; Royal Holloway, University of London, United Kingdom

## Abstract

Previous research suggests that bodily self-identification, bodily self-localization, agency, and the sense of being present in space are critical aspects of conscious full-body self-perception. However, none of the existing studies have investigated the relationship of these aspects to each other, i.e., whether they can be identified to be distinguishable components of the structure of conscious full-body self-perception. Therefore, the objective of the present investigation is to elucidate the structure of conscious full-body self-perception. We performed two studies in which we stroked the back of healthy individuals for three minutes while they watched the back of a distant virtual body being synchronously stroked with a virtual stick. After visuo-tactile stimulation, participants assessed changes in their bodily self-perception with a custom made self-report questionnaire. In the first study, we investigated the structure of conscious full-body self-perception by analyzing the responses to the questionnaire by means of multidimensional scaling combined with cluster analysis. In the second study, we then extended the questionnaire and validated the stability of the structure of conscious full-body self-perception found in the first study within a larger sample of individuals by performing a principle components analysis of the questionnaire responses. The results of the two studies converge in suggesting that the structure of conscious full-body self-perception consists of the following three distinct components: bodily self-identification, space-related self-perception (spatial presence), and agency.

## Introduction

A fundamental endeavour of research on human self-perception is the understanding of the experience of one's own body, i.e., bodily self-perception [1], [2]. An important goal in the study of bodily self-perception is identifying its components when it occurs consciously. This question concerns the structure of conscious bodily self-perception and has so far received little attention. There are a few studies on the structure of conscious *limb* self-perception (e.g., [3]). To our knowledge, however, there are currently no studies published on the structure of conscious *full-body* self-perception.

The induction of the experience of an artificial limb being part of one's own body, i.e., its “embodiment,” is one of the most recent approaches to experimentally investigating bodily self-perception. The rubber hand illusion (RHI; [4]) paradigm belongs to this type of approach and is being used in a growing number of studies. In the RHI, a fake hand being synchronously stroked with the corresponding (but hidden) own hand is perceived as part of one's own body. Longo and colleagues [3] used the RHI to investigate the structure of conscious bodily self-perception. They found that the experiential dimension “embodiment of the rubber hand” was the main aspect of the RHI, and that it could be decomposed into the following three components of bodily self-perception: ownership, i.e., the perception of the rubber hand as part of oneself; location, i.e., the localization of one's own hand or of touch applied to one's own hand in the position of the rubber hand; and sense of agency, i.e., the experience of control over the rubber hand.

The enfacement illusion paradigm is another recently developed experimental procedure used to investigate bodily self-perception. The enfacement illusion consists of a decrease in self-other face discrimination and is induced by the experience of the simultaneous stroking of one's own face along with the face of another person [5], [6]. In accordance with the “ownership” component of the RHI, bodily “self-identification” was identified as one of the experiential components of the enfacement illusion [7].

In order to examine self-perception when experiencing the full body, the RHI procedure has been further developed. This has been achieved by designing experimental setups to induce so-called “full-body illusions.” In these setups, participants wear a head-mounted display in which they see a full illusory or “virtual” body being touched, while they perceive their own physical body being synchronously touched [1], [2]. Full-body illusions are associated with the following changes in bodily self-perception. First, individuals report consciously experiencing the virtual body as their own body [8]–[15], i.e., they experience an alteration of their bodily self-identification. Second, individuals report perceiving changes in their bodily self-localization [16], [17], i.e., they report to perceive themselves in a location other than that of their physical body. This reported alteration of bodily self-localization is accompanied by an increased skin conductance response (SCR) when the virtual body is threatened [16], [17]. In addition, changes found in walking responses [10], in mental imagery [11], in cross-modal perception [8], [12], and in neuronal activity [9] are also regarded as indicating alterations of bodily self-localization. Hence, previous studies on full-body illusions suggest that, corresponding to the components “ownership” and “location” of conscious limb self-perception [3], both “self-identification” and “self-localization” might be distinct components of the structure of conscious full-body self-perception.

A further frequently studied aspect of bodily self-perception is “spatial presence” [18], [19]. Spatial presence refers to the experience of being “there” or present in space with the full body. Hence, spatial presence may be another critical constituent of the structure of conscious bodily self-perception.

Studies on bodily self-identification and self-localization [1], [2], agency [20], [21], as well as spatial presence [18] indicate that these aspects are critical components of conscious full-body self-perception. However, none of the cited studies have investigated the relationship of these aspects to each other, i.e., whether they are distinguishable constituents of the structure of conscious full-body self-perception. Yet, the examination of the relationships between these aspects of full-body self-perception, e.g., the analysis of their correlations, is crucial for determining and, thereby, for understanding the structure of conscious full-body self-perception. Therefore, the objective of our investigation is to elucidate the structure of conscious self-perception when experiencing the full body.

We exposed healthy individuals to a full-body illusion experimental setup in which they saw the back of a distant virtual body being stroked and also perceived a synchronous stroking of their own back. After visuo-tactile stimulation, we asked participants to fill out a custom made questionnaire in which they assessed changes in their bodily self-perception in the abovementioned setup. In order to investigate the structure of conscious full-body self-perception, we analyzed the responses to our questionnaire in a first study using multidimensional scaling combined with cluster analysis. In a second study with the same experimental setup, we then extended the questionnaire and validated the stability of the structure of conscious full-body self-perception found in the first study within a larger sample of individuals by analyzing the questionnaire responses with principle components analysis.

## Materials and Methods

### Ethics statement

Two separate studies were performed. Both studies had the same experimental procedure, which was approved by the local ethics board of the University of Tübingen and was in line with the Declaration of Helsinki.

### Participants

In the first study, twenty-one healthy males (mean age  = 28.9 years, SD  = 5.9 years) and in the second study, fifty-eight subjects (34 females; mean age  = 28.5 years, SD  = 6.6 years), with normal or corrected-to-normal vision participated. All participants gave their written informed consent prior to the experiment.

### Materials and setup

**Figure 1:**
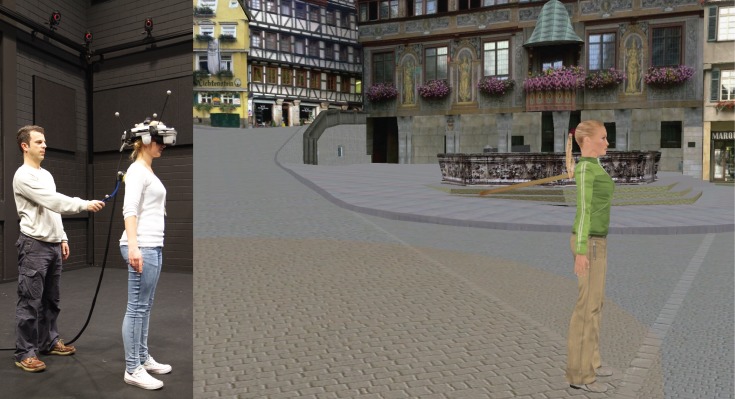
The experimental setup. The experimenter strokes the physical back of a female participant with a motion-tracked stick while she watches, on a head-mounted display, a virtual body (side view) from behind at a distance of 5 meters being synchronously stroked with a virtual stick. The subjects on the photograph have given written informed consent, as outlined in the PLOS consent form, to publication of the photograph.

In both studies, participants wore an Nvis nVisor SX60 head-mounted display (HMD) with a resolution of 1280 by 1024 pixels per eye (in stereo), a refresh rate of 60 Hz per eye, and a diagonal field of view of 60 degrees. The HMD was used for the stereoscopic presentation of a three dimensional “virtual” body on a naturalistic virtual rendering of the marketplace of Tübingen (Figure 1). This was accomplished by means of Virtools 5, using a Dell Precision M6400 computer. Tactile stimulation was provided by a stick. This stick was tracked by a Vicon tracking system consisting of 16 Vicon MX 13 cameras that have submillimeter accuracy in the location where the stroking occurred.

### Procedure

**Figure 2:**
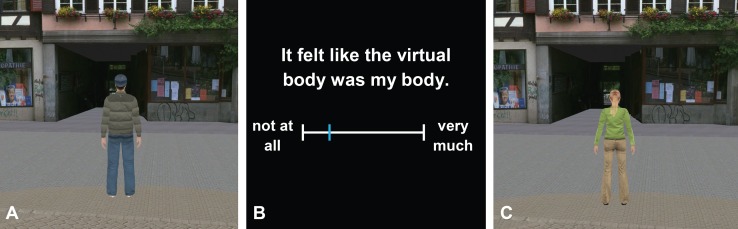
View of the virtual body and of the questionnaire. (A) The view of the male participants when watching the virtual body. (B) The view of the questionnaire (for better visibility, the VAS and the fonts are scaled up) as it was presented on the head-mounted display. (C) The view of the female participants when watching the virtual body.

Standing in an upright position, the participants watched a female or a male virtual body (depending on their gender) from behind at a distance of 5 meters (Figure 2A & 2C) in the HMD. They were asked to take the same neutral standing posture as the presented body and not to move while watching this body. For three minutes, the participants' physical back was stroked with the motion-tracked stick (Figure 1). The tracked motion was applied to a virtual stick, which stroked the back of the virtual body. As a result, the participants saw the back of the virtual body being stroked in synchrony with the motion of the stick that they felt on their back. Immediately after the stroking, the participants assessed their experience with the self-assessment questionnaires described below. In order to mask any external noises, white noise was presented over the built-in headphones of the HMD.

### Self-assessment questionnaire

The participants assessed their experience by retrospectively responding to a set of questionnaire items (Table 1) that were presented in the HMD. These items were formulated as self-report statements, e.g., “It felt like the virtual body was my body.” Below each statement, a visual analogue scale (VAS) was presented. The VAS was a continuous horizontal line of 255 pixels with the left pole labeled “not at all,” and the right pole “very much” (Figure 2B). The participants were instructed to move a small vertical line (see Figure 2B) on the VAS to rate the intensity of the experience described in each statement by using a joystick. For each statement, the VAS was presented such that this vertical line was initially placed at the left pole of the VAS.

**Table 1:** Self-report statements used for the assessment of conscious full-body self-perception.

		Presentation order	
Item	Self-report statement	Study 1	Study 2
1	It seemed as if I might have more than one body.	1	1
2	It felt like I could have moved the head of the virtual body, if I had wanted.	2	2
3	I felt somehow connected with the virtual body.	3	4
4	I experienced the virtual body as a part of myself.	4	5
5	Sometimes, I had the feeling that I was looking at myself.	5	7
6	Sometimes, I had the feeling of standing in the place of the virtual body.	6	8
7	It felt like I was in control of the virtual body.	7	9
8	Sometimes, it felt like I and the virtual body were one.	8	11
9	It felt like the virtual body was my body.	9	12
10	It felt like I could have moved the virtual body, if I had wanted.	10	14
11	It felt like the virtual body belonged to me.	11	16
12	Sometimes, I felt like I was inside the virtual body.	12	17
13	I seemed to feel when the virtual body was touched.	13	19
14	I had the feeling that I was standing in front of myself.	14	20
15	Sometimes, I had the impression that it was me touching myself with the stick.	15	22
16	I had the feeling that I was in the middle of the action rather than merely observing.	16	21
17	I experienced myself as part of the presented environment.	17	3
18	I felt like I was actually there in the presented environment.	18	6
19	I felt like the presented objects were surrounding me.	19	15
20	It was as though my true location had shifted into the presented environment.	20	23
21	It seemed as though I was present in the environment.	21	18
22	I felt as though I was physically located in the presented environment.	22	10
23	It seemed as though I actually took part in the presented action.	23	13
24	It felt like I could have moved the legs of the virtual body, if I had wanted.		24
25	It seemed like the touch I felt was caused by the stick touching the virtual body.		25
26	It felt like I could have moved the arms of the virtual body, if I had wanted.		26
27	It seemed like my body was in the location where the virtual body was.		27

Response format: visual analog scale (minimum  =  not at all; maximum  =  very much).

The statements were in German and described the experience of the virtual body, the experience of the stick touching the virtual body, and the experience of spatial presence. The statements describing the experience of the virtual body included items referring to bodily self-identification, e.g., “It felt like the virtual body belonged to me”; bodily self-localization, e.g., “It seemed like my body was in the location where the virtual body was”; and sense of agency, e.g., “It felt like I could have moved the head of the virtual body, if I had wanted.” Most of the questionnaire items were adapted from previous studies on full-body illusions (e.g., [9],[10],[13]) or from the study on the structure of the RHI [3] described above. The items assessing spatial presence were adapted from the MEC Spatial Presence Questionnaire [19]. Some of the self-report statements were newly formulated in order to ensure that all of the studied aspects of bodily self-perception were well covered.

In the first study, the self-assessment questionnaire consisted of the first 23 items shown in Table 1. The numbers of the self-report statements in Table 1 indicate the order of their presentation in the first study, which was the same for all participants. The 23 items of the first study were also used in the second study and were extended by the last 4 items shown in Table 1. Hence, the questionnaire in the second study consisted of 27 items. As can be seen in Table 1, the presentation order of the first 23 items was slightly changed in the second study, such that the spatial presence items were not all presented together at the end of the questionnaire. In the second study, the item presentation order was also the same for all participants.

### Statistical analyses

In the first study, the structure of the first 23 questionnaire items shown in Table 1 was explored in three steps. First, the responses to each item were pairwise correlated with the responses to each of the other items by calculating Pearson's r. Subsequently, the resulting correlation matrix was analyzed by means of the multidimensional scaling (MDS) algorithm Proxcal in SPSS 20. This algorithm minimizes raw normalized stress. The result of the MDS can be visualized as a common space map on which our self-report statements are represented as points, such that the distances between the points represent the correlations of the responses to the statements. In order to more formally identify clusters of self-report statements that were perceived as similar, we further analyzed the questionnaire responses by means of the ICLUST algorithm [22], [23] using the statistical software R. ICLUST joins any two items or clusters together into a single new cluster if the coefficient alpha [24] and coefficient beta [22] for the new cluster exceed the average coefficient alpha and beta of the two separate items (or clusters) being considered for merging. In the first study, MDS and cluster analysis were used instead of principle components analysis, as they do not have requirements regarding the size of the sample.

In the second study, the structure of the responses to all 27 questionnaire items shown in Table 1 was investigated by performing a principle components analysis (PCA) with varimax rotation using SPSS 20. Moreover, Velicer's minimum average partial (MAP) test [25] was performed according to O'Connor [26]. The MAP test served together with the classic scree test to determine the optimal number of components to be extracted by the PCA.

Sample size is a debated issue in the field of PCA ranging from recommendations regarding the subjects-to-variables ratio to recommendations regarding the absolute minimum number of participants [27]. Although the sample size of our second study falls at the lower end of these recommendations, the adequacy of our PCA is reinforced by the KMO measure and Bartlett's test of sphericity, as well as by the fact that its results replicate those from the first study demonstrating the stability and generalizability of the initial findings.

## Results

### Study 1

**Figure 3:**
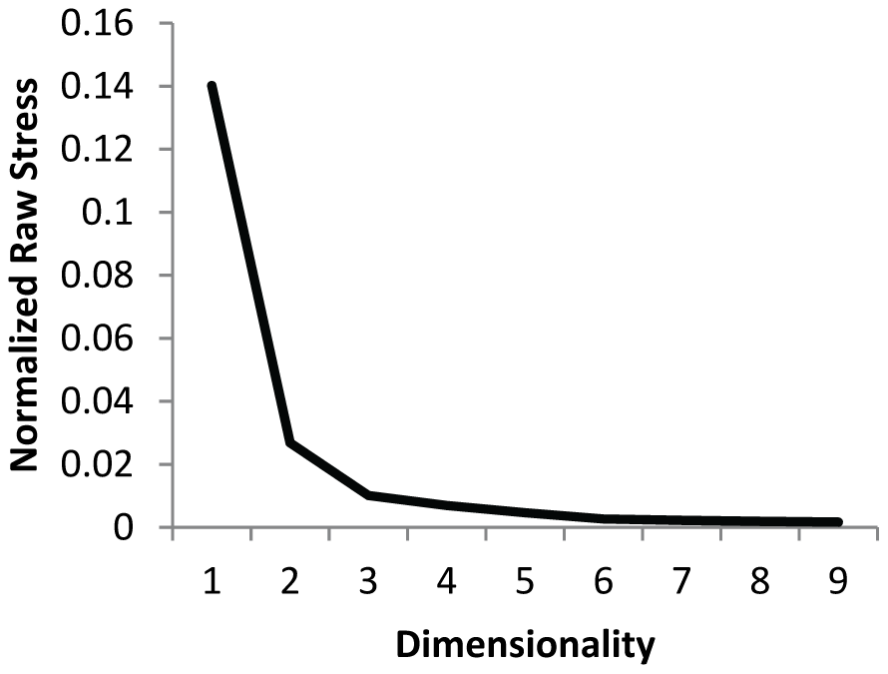
Scree-plot of the normalized raw stress.

The scree-plot of the normalized raw stress of the multidimensional scaling (MDS) of the item responses (Figure 3) in the first study indicated a two-dimensional solution.

**Figure 4:**
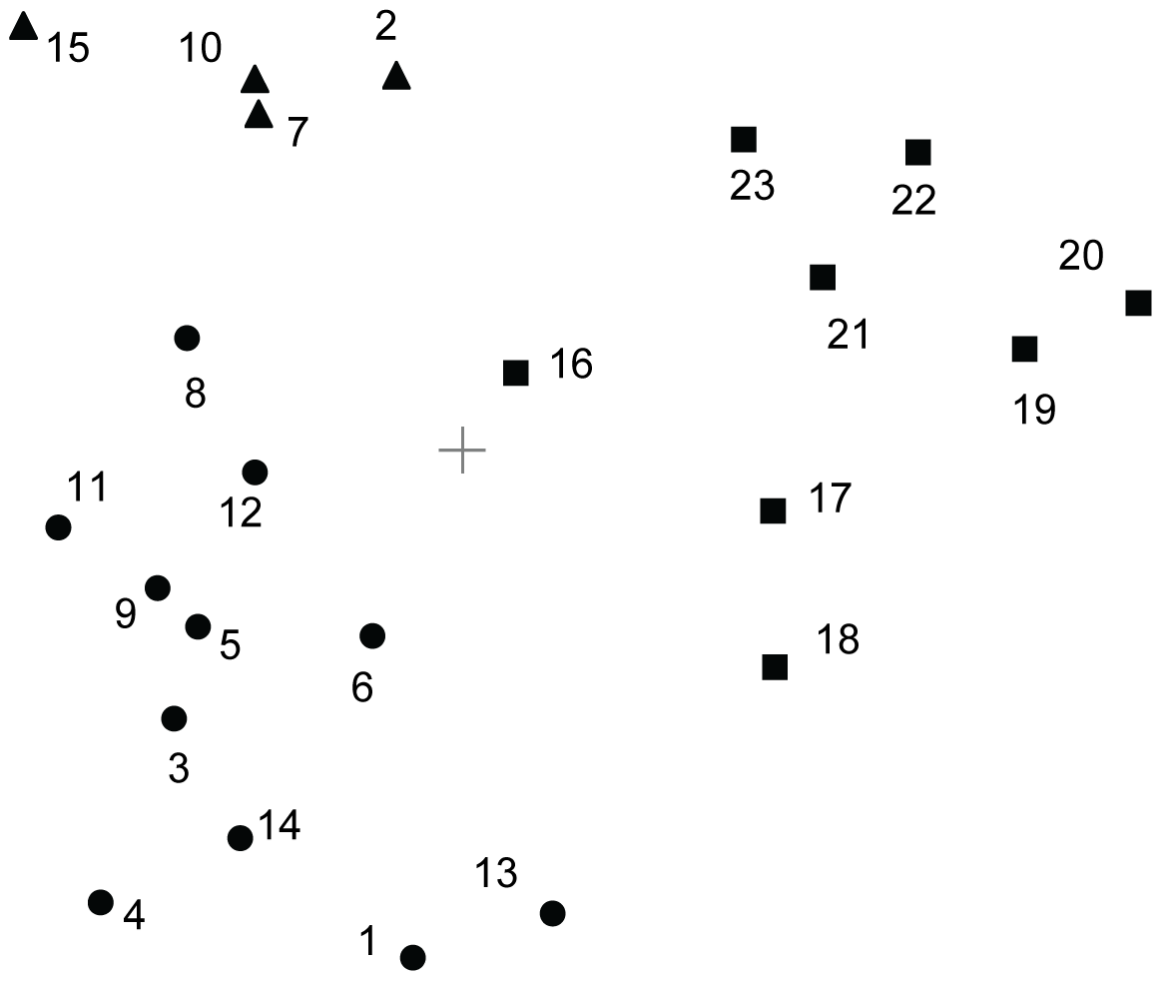
Two-dimensional common space map of the multidimensional scaling of the questionnaire responses in the first study. The numbers indicate the self-report statements shown in Table 1. The symbols indicate that there are three groups of self-report statements: items referring to the experience of *self-identification* with the virtual body (dots); items referring to the experience of *agency* (triangles); and items referring to the experience of *spatial presence* (squares). Normalized raw stress  = 0.026.

Figure 4 shows the visualization of the MDS solution (normalized raw stress = 0.026) as the common space map of the first 23 self-report statements shown in Table 1. This map provides information about the structure of our self-report statements in an easy and intuitive way. Note that, on this map, a small distance between two points indicates a high similarity between the items corresponding to these points. Thus, Figure 4 suggests the emergence of three groups of self-report statements: items related to the illusion of being able to control the virtual body or the stick (agency); items referring to the sense of being present in space (spatial presence); and items referring to the identification with various aspects of the virtual body (bodily self-identification).

**Figure 5:**
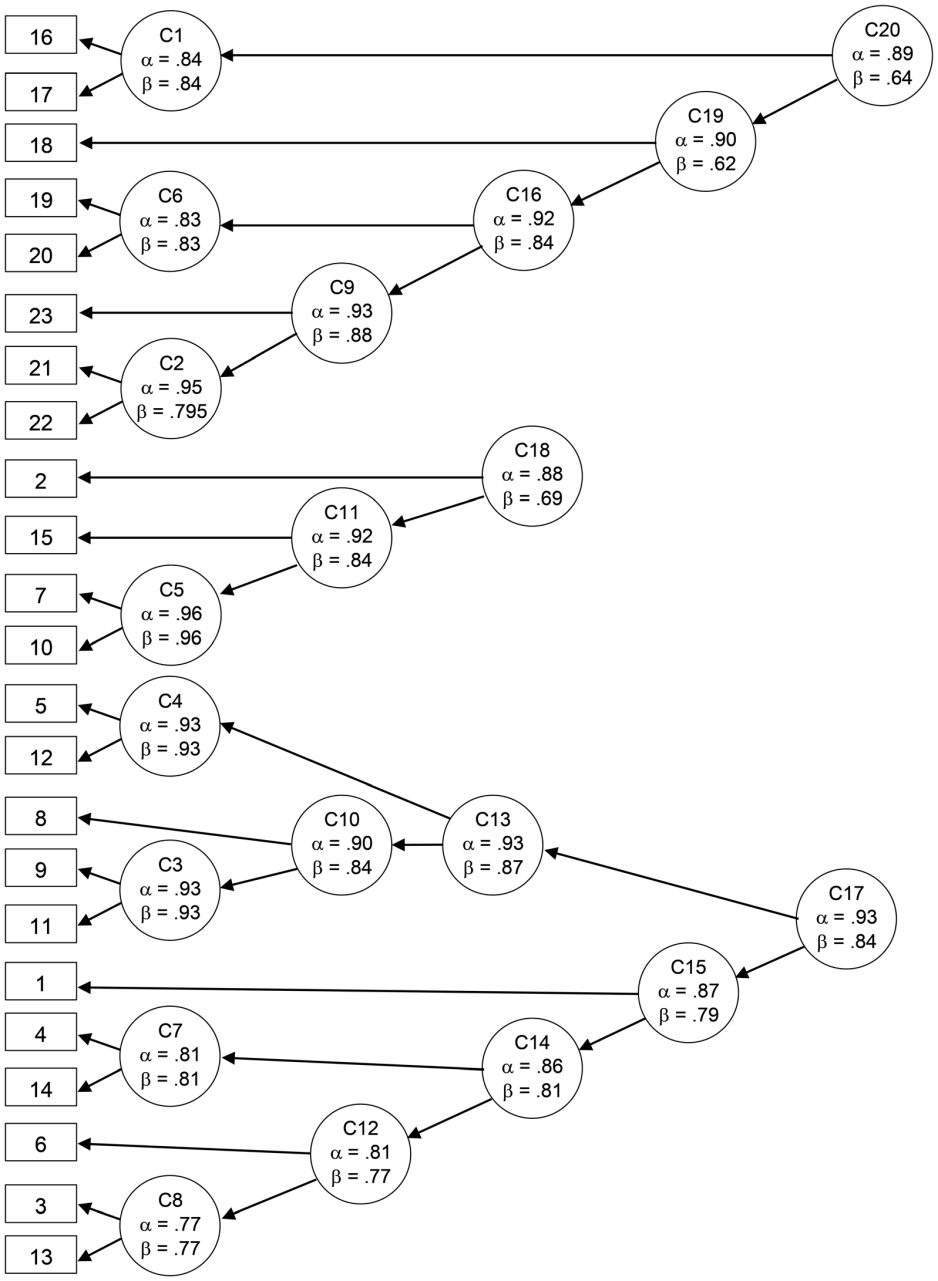
Tree diagram of the cluster analysis (ICLUST). The questionnaire items shown in Table 1 are indicated by numbers within rectangles. There are three higher-order clusters: C17  =  bodily self-identification; C18  =  agency; and C20  =  spatial presence. Cluster fit  =  0.79.

To more formally assess whether the groups of questionnaire items on the MDS map can be found as actual clusters, we performed a cluster analysis by means of the ICLUST algorithm. The dendrogram of the cluster analysis (Figure 5) shows three clusters at the bottom of the cluster tree (cluster fit  = 0.79, pattern fit  = 0.93). The cluster analysis confirms the following three clusters already indicated on the two-dimensional MDS map: a cluster (Cronbach's α = 0.89) consisting of all presence items; a cluster (Cronbach's α = 0.88) consisting of agency-related items; and a cluster (Cronbach's α = 0.93) consisting of items related to the experience of self-identification with the virtual body. Hence, the results of the first study converge in suggesting that conscious full-body self-perception has three basic components. Based on the content (see Table 1) of the items that are part of each of these components (Figure 5), we name them “bodily self-identification,” “spatial presence,” and “agency.”

### Study 2

**Figure 6:**
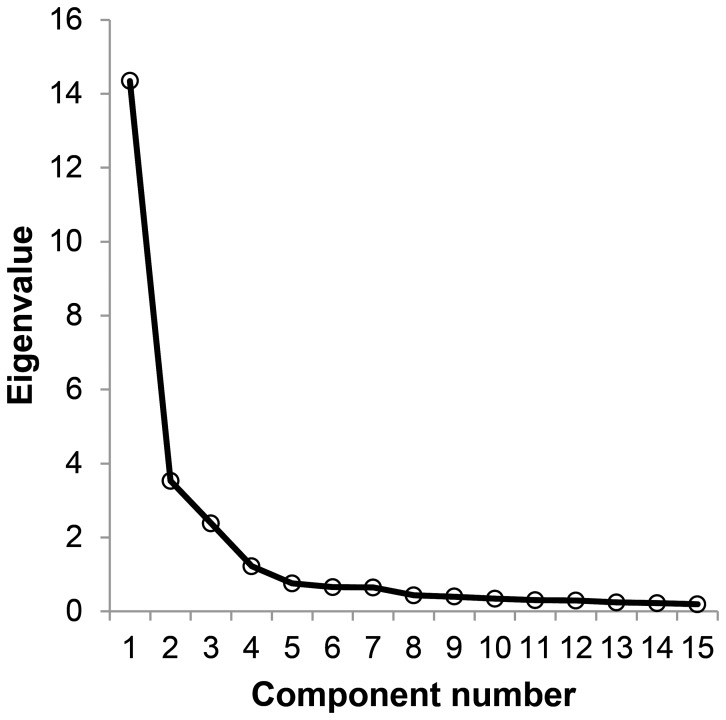
Scree-plot of the eigenvalues.

The Kayser–Meyer–Olkin measure (KMO = 0.880) and the Bartlett's test of sphericity (χ^2^[351] = 1774.9, p = 0.000) indicate the adequacy of performing a principle components analysis (PCA) with the data from the second study. Both the scree test, shown in Figure 6, and the results of the MAP test, shown in Table 2, suggest the extraction of three components.

**Table 2:** Result of the MAP test.

Components partialed out	Squared partial correlation	4^th^ power partial correlation
0	0.2859	0.1197
1	0.0929	0.0261
2	0.0714	0.0155
3	0.0308	0.0027
4	0.0339	0.0037
5	0.0358	0.0051
6	0.0340	0.0050
7	0.0348	0.0046
8	0.0391	0.0053
9	0.0439	0.0064
10	0.0475	0.0075
11	0.0525	0.0088
12	0.0538	0.0081
13	0.0586	0.0097
14	0.0671	0.0134
15	0.0739	0.0151
16	0.0829	0.0195
17	0.0951	0.0235
18	0.1099	0.0322
19	0.1331	0.0450
20	0.1537	0.0563
21	0.1800	0.0728
22	0.2034	0.0927
23	0.2633	0.1336
24	0.3550	0.2268
25	0.5434	0.4179
26	1	1

The average squared, as well as the 4^th^ power partial correlation, are both smallest when 3 components are partialed out of the correlation matrix of the item responses.

Together, the three components account for 75.0% of the variance. As can be seen in Table 3, the items loading on the first component refer to the experience of identification with various aspects of the virtual body or the virtual body as a whole. For example, item 11 “It felt like the virtual body belonged to me” loads on this component. The first component accounts for 53.1% of the variance.

**Table 3:** Result of the principle components analysis of the responses to the 27 self-report statements on conscious full-body self-perception.

		Loading on component			
Item	Self-report statement	C 1: Self-identification	C 2: Spatial presence	C 3: Agency	Commu-nalities
9	It felt like the virtual body was my body.	**.850**	.175	.261	.820
3	I felt somehow connected with the virtual body.	**.846**	.241	.145	.796
12	Sometimes, I felt like I was inside the virtual body.	**.824**	.147	.244	.760
27	It seemed like my body was in the location where the virtual body was.	**.821**	.285	.115	.768
4	I experienced the virtual body as a part of myself.	**.807**	.206	.275	.769
11	It felt like the virtual body belonged to me.	**.798**	.156	.363	.793
8	Sometimes, it felt like I and the virtual body were one.	**.779**	.175	.180	.669
6	Sometimes, I had the feeling of standing in the place of the virtual body.	**.752**	.365	.259	.767
5	Sometimes, I had the feeling that I was looking at myself.	**.740**	.055	.380	.696
25	It seemed like the touch I felt was caused by the stick touching the virtual body.	**.705**	.345	.327	.723
14	I had the feeling that I was standing in front of myself.	**.689**	.035	.424	.655
13	I seemed to feel when the virtual body was touched.	**.626**	.290	.288	.559
1	It seemed as if I might have more than one body.	**.496**	.267	.043	.320
19	I felt like the presented objects were surrounding me.	.046	**.911**	.151	.856
21	It seemed as though I was present in the environment.	.247	**.911**	.167	.919
18	I felt like I was actually there in the presented environment.	.173	**.877**	.142	.819
17	I experienced myself as part of the presented environment.	.167	**.870**	.089	.793
20	It was as though my true location had shifted into the presented environment.	.282	**.862**	.134	.841
22	I felt as though I was physically located in the presented environment.	.370	**.774**	.169	.764
16	I had the feeling that I was in the middle of the action rather than merely observing.	.533	**.646**	.210	.746
23	It seemed as though I actually took part in the presented action.	.577	**.604**	.229	.749
26	It felt like I could have moved the arms of the virtual body, if I had wanted.	.215	.066	**.935**	.925
10	It felt like I could have moved the virtual body, if I had wanted.	.314	.184	**.902**	.946
24	It felt like I could have moved the legs of the virtual body, if I had wanted.	.289	.223	**.874**	.897
7	It felt like I was in control of the virtual body.	.337	.194	**.862**	.895
2	It felt like I could have moved the head of the virtual body, if I had wanted.	.295	.085	**.857**	.829
15	Sometimes, I had the impression that it was me touching myself with the stick.	.124	.192	**.357**	.179

The items loading on the second component refer to the sense of being present in space (spatial presence). For example, item 17 “I experienced myself as part of the presented environment” is part of this component. The second component accounts for 13.1% of the variance. The items loading on the third component refer to the illusion of being able to control the virtual body or the stick. For example, item 26 “It felt like I could have moved the arms of the virtual body, if I had wanted” is part of this component. The third component accounts for 8.8% of the variance.

The loadings and the communalities of item 1 and item 15 are rather small. However, the PCA of the item responses when excluding these two items results in the same three components as described above. Nevertheless, it may be considered to exclude these two items in future studies due to their low communalities.

**Figure 7:**
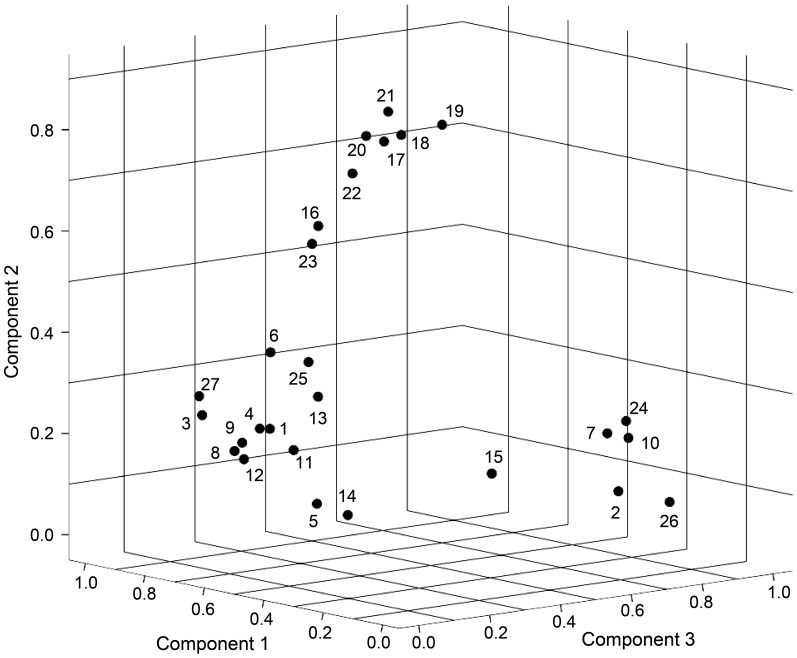
Three-dimensional plot of the loadings of the questionnaire items on the components extracted by the principle components analysis in the second study. The numbers indicate the self-report statements shown in Table 1. The components extracted by the principle components analysis are spanned by a cluster of items referring to the experience of *self-identification* with the virtual body (component 1), a cluster of items referring to the experience of *spatial presence* (component 2), and a cluster of items referring to the experience of *agency* (component 3).

The result of our principle components analysis (PCA) of the responses to the 27 questionnaire items is visualized in Figure 7 as a three-dimensional plot of the loadings of the questionnaire items onto the three components when rotated by the varimax algorithm. As can be seen in Figure 7, the three components are spanned by three clusters of items which fully correspond to the three item clusters found in the first study. Considering the contents (see Table 1) of all items that constitute each of these components (Figure 7), the result of the PCA confirms the finding of the first study that conscious full-body self-perception has three basic components: bodily self-identification (Cronbach's α = 0.96), spatial presence (Cronbach's α = 0.96), and agency (Cronbach's α = 0.94).

## Discussion

We measured conscious experience when perceiving a distant virtual body being simultaneously stroked along with one's own physical body by means of a standardized psychometric self-assessment questionnaire. The objective of our investigation was to explore the structure of conscious full-body self-perception by analyzing the correlations of the responses to the questionnaire items. The findings of our two studies converge in showing that our questionnaire items constitute a structure consisting of three distinct components of bodily self-perception. One of these components is formed solely by questionnaire items taken from the MEC Spatial Presence Questionnaire [19] referring to the sense of presence in space. A second component is spanned by a cluster of questionnaire items referring to the experience of the virtual body as oneself, e.g., item 11 “It felt like the virtual body belonged to me” or item 8 “Sometimes it felt like I and the virtual body were one.” The items belonging to this second component either refer to the identification with the virtual body as a whole or the identification with a partial aspect of the virtual body, e.g., its location. Hence, we termed this component “self-identification.” The third component is defined by questionnaire items measuring the illusion of controlling the virtual body, e.g., item 10 “It felt like I could have moved the virtual body, if I had wanted,” as well as an item referring to the experience of control over the stick. Therefore, we named this component “agency.”

Bodily self-identification and self-localization [28], as well as sense of agency [20], [21] are regarded as crucial aspects of bodily self-perception. As described in the introduction, the experience of the rubber hand illusion (RHI) has been found to involve all of these three aspects [3]. In accordance with this, we find the experience of bodily self-identification with and control over (agency) a distant virtual body as distinct components of full-body self-perception. Hence, our findings suggest that the structure of self-perception during the RHI is partly similar to that during the full-body illusion of experiencing oneself as a distant virtual body. However, in none of our structural analyses did bodily self-localization emerge as a component of full-body self-perception. In fact, our results indicate that questionnaire items on bodily self-localization (e.g., items 6, 13, 25, 27) and those on bodily self-identification refer to the same type of conscious bodily self-experience, as they are part of the same cluster/component. Hence, our findings suggest that bodily self-localization is an aspect of the experience of bodily self-identification.

Previous studies on full-body illusions have not investigated, whether there is a difference between self-localization and self-identification within conscious full-body self-perception; this is because they did not analyze the dependencies of these aspects. Hence, in light of our findings, the question may be raised as to how changes found in previous full-body illusion experiments with measures other than questionnaires shall be interpreted. For example, changes found in skin conductance response following a threat to the virtual body accompanied by individuals reporting perceiving themselves in the location of the virtual body [16], [17] might not reflect the experience of changes of bodily self-localization, but rather of self-identification. Moreover, changes found in walking responses [10], in cross-modal perception [8], mental imagery [11], as well as in neuronal activity [9] when individuals report identifying themselves with a distant virtual body could be interpreted as alterations of self-identification and not specifically of self-localization. Alternatively, it may be speculated that the cited non-verbal measures, which in previous studies were clearly regarded as indicating alterations of conscious bodily self-perception [1], instead indicate changes of bodily self-localization that are not consciously experienced.

It has been suggested that spatial presence is an important aspect of self-perception [18]. Our finding that spatial presence questionnaire items form a distinct component within the structure of full-body self-perception confirms this suggestion. Thereby, our investigation is the first to identify space-related bodily self-perception as one of the basic components of bodily self-perception. Hence, our findings on spatial presence indicate that it is worthwhile to integrate space-related self-perception into a theory of human bodily self-perception.

Based on our findings it would be interesting to investigate the effect of experiencing a virtual arm as part of oneself [29], [30], [31] on spatial presence. Considering that spatial presence occurs and can also be measured in real environments [32], [33], it would also be worthwhile to investigate if it is affected by the RHI. Moreover, by investigating whether spatial presence can be identified within the experience of the RHI or the virtual arm illusion, space-related self-perception may be discovered to be the common basis of limb and full-body self-perception. Therefore, the investigation of the experience of spatial presence during limb illusions, especially during the RHI, can be regarded as an important avenue of future research on conscious bodily self-perception.

In sum, our study suggests that the structure of conscious full-body self-perception consists of three distinct components which we propose to name bodily self-identification, space-related self-perception (spatial presence), and agency. Based on our results, we recommend using the 27 items of our self-assessment questionnaire for the reliable and valid measurement of the abovementioned three components of conscious bodily self-perception within the full-body illusion experimental paradigm.
